# Pharmacokinetic Effect of *MDR* Gene Polymorphism rs2032582 on the Therapeutic Response in Iraqi Patients with Acute Myeloid Leukemia

**Published:** 2020

**Authors:** Rafid A. Abdulkareem, Tamadher Abbas Rafaa, Hamsa Ahmed Jasim, Ahmed Abdul Jabbar Suleiman

**Affiliations:** 1. Institute of Genetic Engineering and Biotechnology for Post Graduate Studies, University of Baghdad, Baghdad, Iraq; 2. University of Anbar, Anbar, Iraq; 3. College of Science, University of Anbar, Anbar, Iraq

**Keywords:** Acute myeloid leukemia, Genetic, *MDR* gene, Polymorphism, Vincristine

## Abstract

**Background:**

The main problem in treatment of leukemia patients is the chemotherapy resistance which is a main concern in recent years. The cause of chemotherapy drug resistance is related to *MDR* gene which is located on chromosome 7 (7q21-31) and it is mainly connected with energy-dependent efflux (P-glycoprotein). This study was conducted to assess the correlation between MDR polymorphism and chemotherapy efficiency with Vincristine in a sample of Iraqi Acute Myeloid Leukemia (AML) patients.

**Methods:**

The blood sample of 200 AML patients and 200 controls were collected and the frequency of rs2032582 was calculated through sequencing and then the role of different genetic patterns was evaluated on cancer cells by MTT assay.

**Results:**

The results indicate that GG and TT genotypes (20 and 20.5% from total patients count) are more frequent in Iraqi AML patients than other genetic patterns in *MDR* gene and also the genotype TA is more sensitive to Vincristine chemotherapy than other genotypes.

**Conclusion:**

It seems that genetic pattern is the main factor in determination of chemotherapy of AML patients, and patients should not undergo chemotherapy with such drugs, especially Vincristine.

## Introduction

Understanding and using pharmacokinetic principles can improve the likelihood of successful therapy and decrease adverse chemotherapy treatment effects in cancer patients ^1^. Many variable factors, whether inherent or obtained, can influence drug disposition and work to confer drug resistance; poor drug solubility, physicochemical characteristics and toxicity to normal tissues restrict the chemotherapy drugs doses while administering to cancer patients whereas pharmacokinetic impacts including distribution, metabolism, absorption, and removal restrict the real quantity of the drug reaching the tumor ^2^.

In addition, several defined mechanisms at the tumor stage confer resistance to one or more chemotherapeutic medications, including debilitated medication take-up inferable from decreased articulation or silence of drug transporters, elevated drug efflux, plasma membrane changes in lipid structure, apoptosis inhibition, increased DNA repair, and cell cycle control ^3,4^.

Multidrug Resistance (MDR) is still a significant barrier to effective cancer chemotherapy ^5^. MDR gene was heavily correlated with cancer susceptibility; it is expressed in cancer cells and in a number of ordinary tissues like intestines, liver, kidneys, blood-brain barriers, spinal cord and placenta. Adverse effect of drug resistance after chemotherapeutic treatment is associated with over-expression or *MDR1* amplification ^6^.

*MDR1* gene has a length of about 200 *kb* containing 29 exons and lies on the long arm of the human chromosome 7q21; this gene codes for P-glycoprotein (P-gp) efflux transporter which limits and deposits a broad range of medications from cells into the extracellular space. P-glycoprotein thus helps to withstand the impacts of chemotherapy drugs on cancer cells ^7^.

It has recently been shown that *MDR1* is an extremely polymorphic gene and has been recognized with various mutations; in fact, 38 Single Nucleotide Polymorphisms (SNPs) were identified in the coding region of 50 reported *MDR1* polymorphisms. The existence of polymorphisms in this gene can lead to gene expression, amino acid sequence in protein composition, protein functions, and therapy reaction ^8,9^.

The present study, therefore, focused on the potential association of G2677T T *MDR1* gene polymorphism with Acute Myeloid Leukemia (AML) and its effect on treatment with Vincristine drug in Baghdad Province in Iraq.

## Materials and Methods

### Ethics statement

The research protocol was approved by the Ethics committee of University of Baghdad, and all respondents received full informed consent.

### Study population

The present survey was conducted in Al-Yarmouk Teaching Hospital in Baghdad, Iraq with 200 patients including 122 males and 88 females (Median age of 39.38±6.24 years, range of 35–68) from October 2018 to June 2019 with AML diagnosis. According to normal morphological and immunophenotypic criteria, 200 individuals (77 males and 133 female, matched unrelated in age and sex, disease-free) were regarded as the control group.

### Collection of blood samples and isolation of genomic DNA

For later extraction of DNA, a total of 2 *ml* venipuncture peripheral blood specimens were obtained from all patients and controls. DNA was extracted manually from both using the particular technique earlier mentioned by Qiagen extraction kit (Qiagen, Hilden, Germany) according to the manufacturer's instructions. NanoDrop1000 (Thermo Scientific, USA) was used for the amount of DNA output.

### Genotyping of MDR gene

The G2677T polymorphism (rs2032582) of the *MDR* gene was analyzed using gold standard sequencing method. The primer sequences used in the reaction were F: 5′TCAGCATTCTGAAGTCATGGAA-3 and R: 5′-TTAGAGCATAGTAAGCAGTAGGGAGT-3 primers and they were designed for this study using online tool (http://www.bioinformatics.nl/cgi-bin/primer3plus/primer3plus.cgi/).

The combination of the PCR reaction included 1.5 *μl* of DNA, 2 *μl* of each primer, 15 *μl* of Taq master mix, to bring the final volume to 25 *μl*. Next, PCR grade water was added. The conditions for PCR were as follows: denaturation at 94 °*C* for 5 *min*, 30 denaturation cycles at 95 °*C* for 2 *min*, annealing at 56 °*C* for 1 *min* and extension at 74 °*C* for 1 *min*, and then extension for 10 *min* at 74 °*C*. The amplification was performed with Thermal Cycler (Applied Biosystems™). The obtained PCR amplicon was separated at 140 V by electrophoresis on a 2% agarose gel for 90 *min* and analyzed under ultraviolet light after staining with GelRed (UVP Visi-Blue™, Fisher Scientific).

### Chemosensitivity assay

***Cells:*** Ten *ml* of heparinized peripheral blood from patients with acute leukemia were obtained. Blast cells were separated by centrifugation of the ficoll hypaque density gradient. The isolated cells were then washed twice in RPMI 1640 culture medium supplemented with 10% fetal calf serum. The cell viability was determined by exclusion of trypan blue dye. Cell numbers were adapted for the MTT assays after counting. The samples contained at least 90 leukemic blast cells.

### Drugs and treatment

Sigma Aldrich (Germany) provided Vincristine to assess the impact of drug alternatives from Vincristine stock (1.0 *μg/ml*) in Phosphate-Buffered Saline (PBS) and it was stored at −20 °*C*. Drug-treated groups were treated 4 *hr* before harvesting with the respective drug.

### MTT assay

To assess the viability of cells, MTT assay was used with minor modifications. At first, 50 *μl* of cell suspension containing 2×105 cells/well were seeded in 96-well microtiter plates and Vincristine in a volume of 50 *μl* has been added to each well. Next, 25 *μl* of MTT solution (1*mg/ml* final concentration) was introduced to each well. And for a period of 5 *hr*, the plates were returned to the incubator. Thereafter, the medium was removed and to dissolve the crystals formation, 200 *μl* of dimethyl sulfoxide was added from each well.

The absorbance at 540 *nm* wavelength was measured after 72 *hr* of incubation at 37 °*C* using a microplate reader (Organon Teknika Reader 530). Untreated wells (cells incubated in the culture medium alone) were used as a cell viability control. Vincristine concentration needed to decrease the absorption to 50% of the control at 540 *nm* was chosen as the sample's ID50.

### Statistical analysis

Data was analyzed statistically by SPSS version 22.0 (Armonk, New York, 2013). The genotype frequencies were compared in both groups using the precise Fisher test and mean±SD of ages were calculated in both groups. The Confidence Interval of 95 percent (95% CI) and the odds ratio were calculated between groups. Significant results were assumed when p≤0.05.

## Results

Polymorphism of the *MDR1* gene (G2677 T) was successfully genotyped for all collected blood samples. Medical characteristics (Sex, mean age, family history, smoking) for patients and healthy individuals are listed in table 1.

**Table 1.** The main selected parameters of the patient group and healthy group

**Characteristics**	**Patients with AML**	**Control**
**Sample size**	200	200
**Sex**		
**Male**	134	100
**Female**	66	100
**Ages**		
**Minimum**	35	25
**Maximum**	61	54
**Mean±SD**	38.20000±11.71674	31.65833±11.49167
**Family history**		
**Positive**	74	
**Negative**	126	

The mean age±SD in patients was 38.20000±11.71674, while the mean age±SD of control group was 31.65833±11.49167. The results also indicate that 35 patients had an AML history in their family, while 165 patients had no AML family history. It was found that the G allele is more frequent in the present study. Overall, frequencies of G2677 T genotypes GG, GT, GA TT, and TA were 40(20%), 34(17%), 29(14.5%), 41(20.5%), and 56(28%), respectively in the patient group as shown in table 2.

**Table 2.** Allele frequency and genotype distribution for SNPs in *MDR* gene in leukemia patients and the controls

***MDR*gene polymorphism (rs2032582)**	**Cases l**		**Control**		**OR**	**(95% CI)**	**Fisher's exact test**

	**No.**	**%**	**No.**	**%**			
**GG**	40	20	103	51.5	0.88	(0.55–1.25)	p=0.001^**^
**GT**	34	17	51	25.5	0.25	(0.12–0.55)	p=0.601
**GA**	29	14.5	25	12.5	0.89	(0.45–1.65)	p=0.602
**TT**	41	20.5	13	6.5	1.05	(0.57–2.00)	p=0.614
**TA**	56	28	8	4.0	1.07	(0.41–2.67)	p=0.0082^**^
**Total**	200	100	200	100			

OR: Odds Ratio, CI: Confidence Interval, p: Level of statistical significance, * Statistically significant differences (p<0.05).

Also, in patients, the results of family history correlation with genotypes reveal that 74 patients had a history of infection in their families (AML), while 126 patients had no family history of AML as illustrated in table 3.

**Table 3.** The correlation between distribution of genotype among patients and family history

**Family history**		**Genotype**					**Total**	**(95% CI)**		**Fisher's exact test**
	
		**GG**	**GT**	**GA**	**TT**	**TA**		Lower bound	Upper bound	
**Positive**	Count	22	24	19	5	4	74	(0.344313–0.464538)		p=0.025
	Total %	11%	12%	9.5%	2.5%	2%	37%			
**Negative**	Count	18	10	10	36	52	126			
	Total %	9%	5%	5%	18%	26%	63%			
**Total**	Count	40	34	29	41	56	200			
	Total %	41%	13%	17.5%	9%	19.5%	100%			

A high statistical significance (p=0.025) of 95% CI (0.344313–0.464538) was shown in our results, whereas the frequency of GG, GT, GA, TT and TA genotypes between patients with family history (RA) was as follows: 22(11%), 24(12%), 19(9.5%), 5(2.5%), and 4(2%), respectively. By comparison, the prevalence of GG, GT, GA, TT and TA genotypes between patients with no family history (AML) was as follows: 18 (9%), 10(5%), 10(5%), 36(18%) and 52(26%).

The statistical correlation between Vincristine chemotherapeutic effectiveness and the distribution of genotypes between patients shows significant difference (p=0.029) with 95% CI (0.004526–0.0395627).

In addition, the frequency of GG, GT, GA, TT and TA genotypes among patients treated with Vincristine (Responders) chemotherapy was 10(5%), 11(5.5%), 19(9.5%), 33(16.5%) and 49(24.5%) respectively. GG, GT, GA, TT and TA genotype frequency among patients with negative chemotherapy response to Vincristine (Nonresponses) was as follows: 30(15%), 23 (11.5%), 10(5%), 8(4%) and 7(3.5%) as shown in table 4.

**Table 4.** Distributions of genotypes and alleles of *MDR1* gene polymorphism in relation to chemotherapeutic efficacy of Vincristine

**Chemotherapeutic efficacy**		**Genotype**					**Total**	**(95% CI)**		**Fisher's exact test**
	
		**GG**	**GA**	**GT**	**TT**	**TA**		**Lower bound**	**Upper bound**	
**Responders**	Count	10	11	19	33	49	122	(0.004526–0.0395627)		p=0.029
	Total %	5%	5.5%	9.5%	16.5%	24.5%	61%			
**Nonresponses**	Count	30	23	10	8	7	78			
	Total %	15%	11.5%	5%	4%	3.5%	39%			
**Total**	Count	40	34	29	41	56	200			
	Total %	20%	17%	14.5	20.5%	28%	100%			

## Discussion

SNP is a typical polymorphism structure in human legacy. DNA polymorphisms brought about by SNP may realize singular contrasts in vulnerability to malignancies. A few examinations show that the *MDR1* quality has a few SNPs, which are identified with the advancement of threatening tumors, for example, bosom malignant growth, leukemia, colorectal disease, and glioma ^10^. Hemauer *et al* discovered that a few SNPs were related with a diminished degree of P-gp protein, while the other is connected with an expansion in P-gp transport movement ^11^. In the meantime, GG genotype of G2677T/A was seen to be connected with the most significant level of *MDR1* and AT was for the least level ^12^. A low degree of *MDR1* was displayed in gastric disease cell lines ^10^. *In vitro* analysis showed that knockdown of *MDR1* could clearly build the affectability to Adriamycin treatment ^13^. Further examination proposed that P-gp protein as an oncofetal protein in cells of gastric malignancy could advance cell endurance ^14^.

Articulation of the P-gp efflux siphon diminishes intracellular harmful medication levels and in this manner reduces apoptosis. It accounts for those hereditary *MDR1* polymorphisms that influence the articulation and capacity of efflux pump in sound volunteers ^15^. Subsequently, another part of modified viability of treatment might be P-gp variable function, inferable from the nearness of different genotypes of *MDR1* gene in AML patients. These variations couldn't just impact affectability or obstruction; they could likewise impact treatment result by altered drug clearance ^16,17^.

There are a few reports about explicit components of the up-regulation of the *MDR1* transcript, decreased level of methylation of the *MDR1* as an effector for up-regulation of *MDR1* gene in patients with AML, and quality improvements for *MDR1* articulation ^18^. It appears to be significant that AML patients with the CC genotype may harbor progressive cytogenetic low-risk abnormalities ^19^ which may have a positive effect on *MDR1* expression regulation ^20^ Furthermore, patients with the C variant(s) have higher expression rate of *MDR1* gene. At present, it isn't known how polymorphisms in *MDR1* gene impact quality articulation. The C variation in exon 21 of the *MDR1* gene is responsible for changed *MDR1* protein ^9^. This variation represents amino acid change in alanine to serine at codon 893 of P-gp and is liable for optimal function of protein ^21^.

One potential contender for a useful linkage is the variation at position 129 of the *MDR1* gene that was shown to be related with the decrease in P-gp articulation ^22^. Since this sequence of *MDR1* is not related to the G-box, CAAT box, and responsive components, the exact useful mechanism of this polymorphism remains to be resolved. In many cases, most researched mutations with components of *MDR1* show that one genotype, homozygous A variation, is related with a decrease of *MDR1* articulation in AML patients. Besides, AML patients who have these variations show the most reduced *MDR1* mRNA expression. Additionally, consolidated B genotype heterozygous variations of the *MDR1* gene tend to be phenotypically related to higher or average levels of *MDR1* gene expression. Subsequently, assurance of connected polymorphisms is of worth on the grounds that polymorphic regions may include diverse *MDR1* articulation after introduction to the cytotoxic medications ^22^. In this light, it appears to be significant that heterozygous cases express both alleles of *MDR1* gene as it could be appeared for the B variation in exon 21. In this manner, inactivation of *MDR1* doesn't happen in patients with optimal articulation and in patients with heterozygous genotype. A case of such modifications could be transformations of p53 tumor suppressor.

## Conclusion

This work revealed that *MDR* gene polymorphism may affect the decision for chemotherapy and the family history with the specific genetic pattern may be considered a risk factor for AML distribution.
